# Response surface methodology mediated optimization of phytosulfokine and plant growth regulators for enhanced protoplast division, callus induction, and somatic embryogenesis in *Angelica Gigas* Nakai

**DOI:** 10.1186/s12870-024-05243-w

**Published:** 2024-06-11

**Authors:** Han-Sol Lee, Jong-Eun Han, Eun-Kyung Bae, Eun Yee Jie, Suk Weon Kim, Hyuk Joon Kwon, Hak Sung Lee, Soo-Ho Yeon, Hosakatte Niranjana Murthy, So-Young Park

**Affiliations:** 1 Department of Horticultural Science, Division of Animal, Horticultural and Food Sciences, Chungbuk National University, Cheongju, 28644 Republic of Korea; 2 Department of Forest Bioresources, National Institute of Forest Science, 39 Onjeong-ro, Suwon, 16631 Republic of Korea; 3 Biological Resource Center, Korea Research Institute of Bioscience and Biotechnology (KRIBB), Jeongeup, 56212 Republic of Korea; 4 Food Science R&D Center, Kolmar BNH Co., Seocho-gu, Seoul, 30003 Republic of Korea; 5 Department of Botany, Karnatak University, Dharwad, 580003 India; 6 Department of Biotechnology, KLE Technological University, Hubballi, 580039 India

**Keywords:** Response surface methodology, Protoplast culture, Angelica gigas, Somatic embryogenesis, Phytosulfokine

## Abstract

**Background:**

*Angelica Gigas* (Purple parsnip) is an important medicinal plant that is cultivated and utilized in Korea, Japan, and China. It contains bioactive substances especially coumarins with anti-inflammatory, anti-platelet aggregation, anti-cancer, anti-diabetic, antimicrobial, anti-obesity, anti-oxidant, immunomodulatory, and neuroprotective properties. This medicinal crop can be genetically improved, and the metabolites can be obtained by embryonic stem cells. In this context, we established the protoplast-to-plant regeneration methodology in *Angelica gigas*.

**Results:**

In the present investigation, we isolated the protoplast from the embryogenic callus by applying methods that we have developed earlier and established protoplast cultures using Murashige and Skoog (MS) liquid medium and by embedding the protoplast in thin alginate layer (TAL) methods. We supplemented the culture medium with growth regulators namely 2,4-dichlorophenoxyaceticacid (2,4-D, 0, 0.75, 1.5 mg L^− 1^), kinetin (KN, 0, 0.5, and 1.0 mg L^− 1^) and phytosulfokine (PSK, 0, 50, 100 nM) to induce protoplast division, microcolony formation, and embryogenic callus regeneration. We applied central composite design (CCD) and response surface methodology (RSM) for the optimization of 2,4-D, KN, and PSK levels during protoplast division, micro-callus formation, and induction of embryogenic callus stages. The results revealed that 0.04 mg L^− 1^ 2,4-D + 0.5 mg L^− 1^ KN + 2 nM PSK, 0.5 mg L^− 1^ 2,4-D + 0.9 mg L^− 1^ KN and 90 nM PSK, and 1.5 mg L^− 1^ 2,4-D and 1 mg L^− 1^ KN were optimum for protoplast division, micro-callus formation and induction embryogenic callus. MS basal semi-solid medium without growth regulators was good for the development of embryos and plant regeneration.

**Conclusions:**

This study demonstrated successful protoplast culture, protoplast division, micro-callus formation, induction embryogenic callus, somatic embryogenesis, and plant regeneration in *A. gigas*. The methodologies developed here are quite useful for the genetic improvement of this important medicinal plant.

## Background

Protoplast isolation, culture, and plant regeneration are useful techniques for obtaining single-cell clones, genetic transformation, and producing transgenic plants [1, 2]. Protoplast fusion opens up new avenues in overcoming the barriers of sexual reproduction and increasing the variability among cultivated plants by nuclear and cytoplasmic gene transfer [3, 4]. In addition, insertion, deletion of genes, and gene editing are possible through the transient transformation of protoplasts and by the use of clustered regularly interspaced short palindromic repeat (CRISPR)/CRISPR-associated protein 9 (Cas9) technologies [5]. Furthermore, protoplasts are valuable tools in the areas of molecular biology, genetics, and physiological studies [1, 2].

*Angelica gigas* Nakai is a short-lived perennial plant that is popularly called ‘Purple parsnip’ and is an important medicinal plant that is naturally occurring in China, Japan, and Korea. It is reported to have several pharmacological activities such as anticancer [6, 7], antidiabetic [8], anti-inflammatory [9], anti-platelet aggregation [10], antimicrobial [11], anti-obesity [12], anti-oxidant [13], immunomodulatory [14] and neuroprotective [15] effects. Coumarins namely decurisin, decursinol, and decursinol angelate are bioactive compounds that are responsible for the majority of biological activities [16]. In recent years, this plant has been utilized in the cosmetic industry since it was reported to have anti-wrinkle and treatment of skin photoaging [17, 18].

While many plants have undergone protoplast isolation, culture, and plant regeneration, it can be exceedingly challenging to develop a protoplast-to-plant system in some plants. Various factors such as source material used in protoplast isolation, culture medium, method of culture (liquid or semi-solid, or their combination), plant growth regulators (auxins, cytokinins, and their combination), plating density, supplements added to culture medium including polyamines, growth peptides are affecting a lot during protoplast division, micro-callus formation, and plant regeneration [1]. Recently, we were involved in protoplast isolation from embryogenic callus and plant regeneration via somatic embryogenesis in *Angelica gigas* [19] and evaluated the effect of the enzyme incubation period, culture method, and culture medium. However, the frequency of protoplast division was very low. Recently, PSK, a peptidyl growth factor, has been utilized by several researchers to promote cell division, proliferation, and dedifferentiation in protoplast cultures of *Beta vulgaris*, *Brassica oleracea* var. *capitata*, *Daucus* species, *Fagophyrum tataricum*, and *Nicotiana benthamiana* [20–25]. PSK is reported to be involved in other physiological responses including the promotion of cell growth, development, reproduction, somatic embryogenesis, and plant regeneration [26]. Therefore, in the current study, we utilized PSK, in addition to 2,4-D and KN for induction of cell division, micro-callus formation, and induction of embryogenic callus in *Angelica gigas* protoplast cultures. The statistical method, RSM was described as an excellent tool for exploring the relationships between controllable factors and important response variables [27]. This methodology has been recently applied to biological experiments to evaluate key factors that can optimize specific experimental conditions including cell and organ cultures [28–30]. Therefore, in the present study, RSM was applied to evaluate the influence of 2,4-D, KN, and PSK levels on the induction of protoplast division, microcolony, and embryogenic callus formation with protoplast cultures of *Angelica gigas*.

## Results

### Analysis of protoplast for growth, and development

**Fig. 1:**
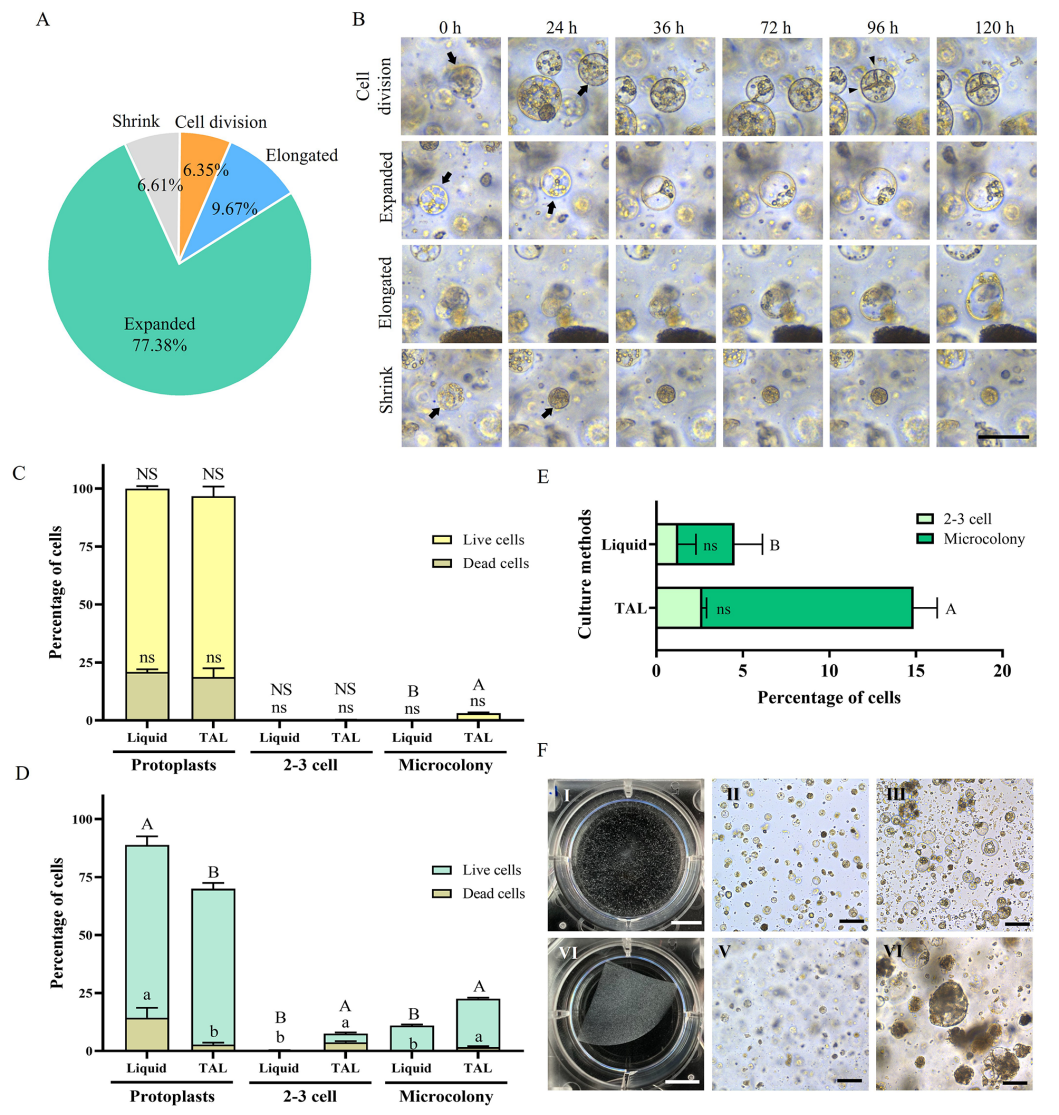
**A-B.** Tracking of the protoplast division of *Angelica gigas* using a compound microscope from 0 to 120 h/5 days: protoplasts were embedded by the TAL method and cultured in MS liquid medium supplemented with 0.3 M mannitol, 0.08 M sucrose, and 0.5 mg L^− 1^ 2,4-D. The cultures were maintained in the dark. Percentage of expanding, shrunken, elongated, and dividing protoplasts (**A**), images of dividing, expanded, elongated, and shrunken protoplasts over the culture period (**B**). Scale bar = 50 μm. **C-E**. Protoplast culture efficiency of *Angelica gigas* in liquid and TAL medium. The cultures were maintained in the dark. Percentage of cell division after 3 weeks of culture (**C**), after 5 weeks of culture (**D**), and after 7 weeks of culture (**E**). Protoplast culture in liquid cultures (**F**), freshly isolated protoplast (**I**, **II**), and after 3 weeks (**III**). Protoplast culture in TAL cultures (**VI**), freshly embedded protoplast in TAL (**V**), and micro-callus formed by the division of protoplasts TAL culture (**VI**). Scale bars: 100 μm (black bars) and 1 cm (white bars)

In the initial experiment, we embedded protoplasts in a TAL, and TAL sections were cultured in MS liquid medium supplemented with 0.4 M mannitol, 0.08 M sucrose, and 0.5 mg L^− 1^ 2,4-D, and the cultures were maintained in the dark. Observations were made on the growth, development, and division of protoplasts at intervals of every 24 h, up to 120 h (5 days), and the data is presented in Fig. 1A and B. Among the protoplasts cultured 77.38% of the protoplasts involved their expansion (Fig. 1A and B), however, such protoplasts were not involved in division even with a lapse of time. Only 6.35% of protoplasts involved division and division starts after 36 h onwards (Fig. 1A and B). 9.67% of cells involved cell elongation and 6.61% of cells were involved in shrinkage (Fig. 1A and B).

### Protoplast culture and comparison of liquid and TAL cultures

Protoplasts were initially cultured by using a liquid medium and the TAL method and assessed their performance on division and microcolony formation at different intervals of time viz. 3rd, 5th up to 7 weeks, and data is presented in Fig. 1C-E. Three weeks after the culture 79.04% of protoplasts were viable in liquid cultures, however, cells were not involved in any division. In contrast, with TAL cultures 78.07% of cells were viable and 0.28% were in the 2–3 celled stage and 3.07% were in involved microcolony formation (Fig. 1C). After, 5 and 7 weeks again 3.73% and 2.66% cells were in the 2–3 celled stage, and 20.89 and 12.20% were in microcolony stages with TAL cultures (Fig. 1D and E). The images of liquid and TAL cultures are presented in Fig. 1FI-III and Fig. 1FIV-VI respectively. The protoplasts cultured in TAL cultures were involved in division and formed micro-calli (Fig. 1FVI).

### Application of RSM to optimize the 2,4-D, KN, PSK on cell division, microcolony formation, and embryogenic callus induction

**Fig. 2:**
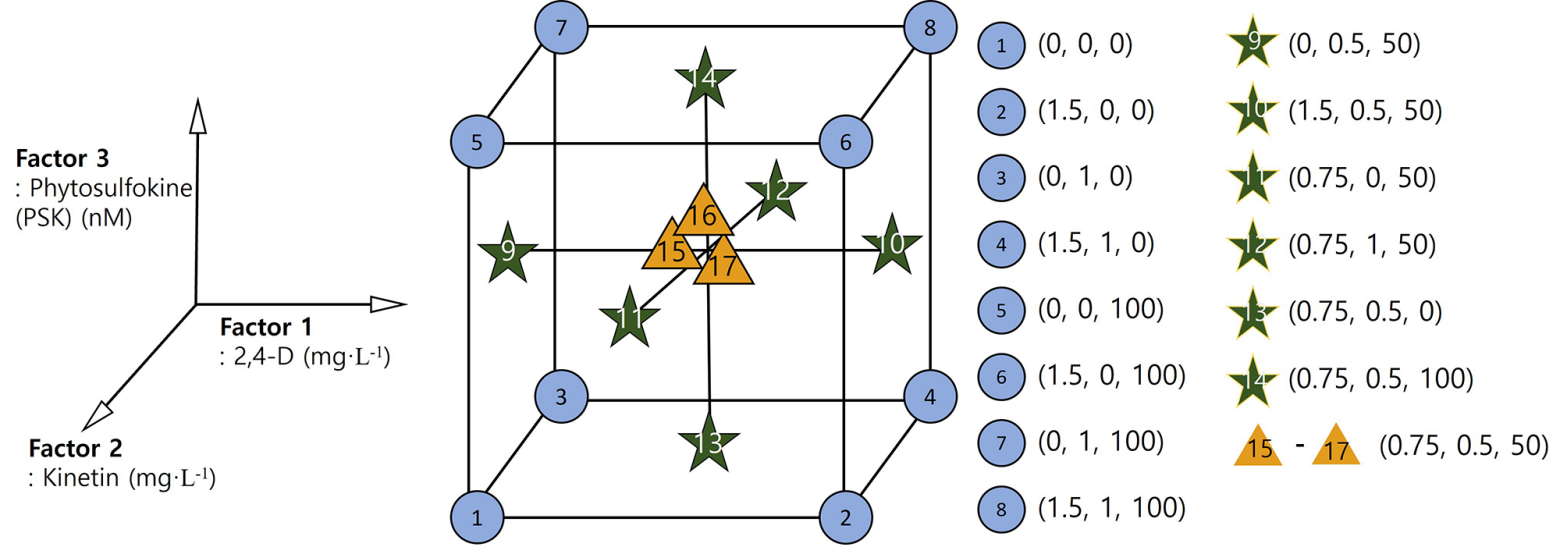
CCD for the verification of effect 2,4-D, KN, and PSK on protoplast division, micro-callus formation, and induction of somatic embryogenic callus during protoplast culture using RSM.

Based on the preliminary results, we decided to culture the protoplasts using the TAL method to verify the impact of 2,4-D (0, 0.75, 1.5 mg L^− 1^), KN (0, 0.5 and 1.0 mg L^− 1^), and PSK (0, 50, 100 nM). We adopted RSM to evaluate the effects and to predict the suitable concentrations of 2,4-D, KN, and PSK. Factor 1 (2,4-D), factor 2 (KN), and factor 3 (PSK) central composite design (CCD) points are shown in Table 1; Fig. 2. This factorial design was applied at three different stages to assess effect factors on protoplast division after 1 week, to evaluate their effect on micro-callus formation after 4 weeks, and to assess their effect on the formation of embryogenic callus after 12 weeks of culture.

**Table 1:** Central composite design points for the protoplast culture of *Angelica gigas*

Design points	Space Type	Factor 1	Factor 2	Factor 3
		2,4-D	KN	PSK
		mg∙L^− 1^	mg∙L^− 1^	nM
1	Axial	1.5	0.5	50
2	Factorial	1.5	0	0
3	Axial	0	0.5	50
4	Factorial	0	1	0
5	Factorial	0	0	0
6	Factorial	1.5	0	100
7	Factorial	1.5	1	0
8	Factorial	1.5	1	100
9	Center	0.75	0.5	50
10	Center	0.75	0.5	50
11	Center	0.75	0.5	50
12	Axial	0.75	0.5	100
13	Axial	0.75	1	50
14	Factorial	0	1	100
15	Factorial	0	0	100
16	Axial	0.75	0	50
17	Center	0.75	0.5	50
18	Axial	0.75	0.5	0

**Fig. 3:**
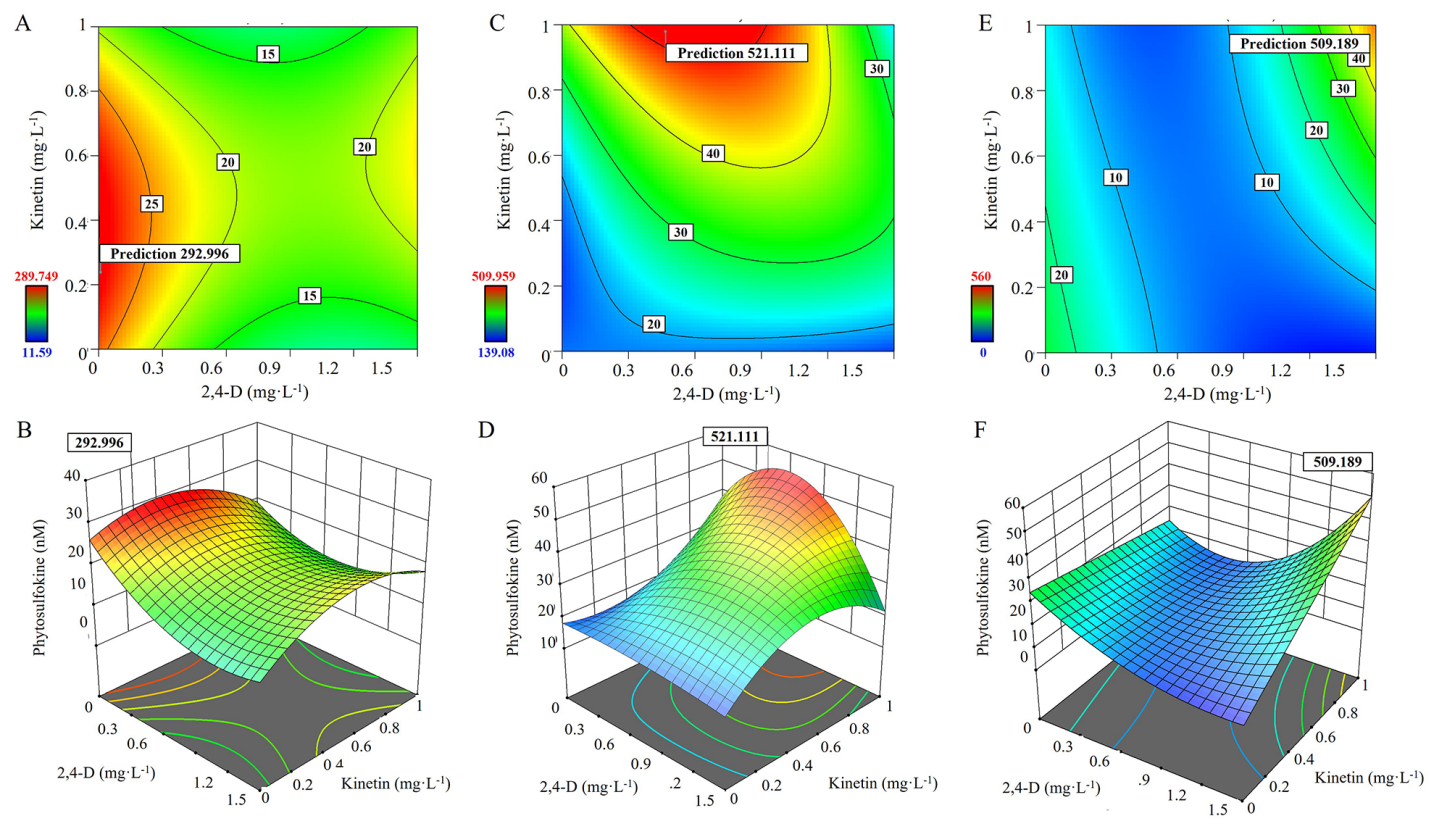
Contour and 3D surface plots showing the effects of 2,4-D, KN, and PSK on protoplast division after 1 week (**A** and **B**), after 4 weeks (**C** and **D**), and after 12 weeks (**E** and **F**) respectively

Table 2 summarizes the findings of the analysis of variance (ANOVA) for protoplast division. The finding showed that the three components, 2,4-D, KN, and PSK, had a linear relationship. In addition, interaction 2,4-D × KN, 2, 4-D × PSK, and 2,4-D × KN × PSK and their varied concentrations showed quadratic effects. All the interactions 2,4-D, KN, and PSK, 2,4-D × PSK and KN × PSK, and 2,4-D × KN × PSK depicted statistically significant results. Figure 3A and B showed a 2D contour plot and 3D response surface plots for the protoplast division. These results indicated that optimal concentrations for the protoplast division are 0.04 mg L^− 1^ 2,4-D + 0.5 mg L^− 1^ KN + 2 nM PSK. The results demonstrated that the interaction of PSK along with 2,4-D and KN enhanced the cell division of TAL-cultured protoplasts of *A. gigas*.

**Table 2:** ANOVA table for the effect of 2,4-D, KN, and PSK on the frequency of cell division of *Angelica gigas* protoplasts after one week of culture

Source	Sum of Squares	df	Mean Square	F value	*p* value Prob > F	Significance
Model^a^	2605.09	11	236.83	16.92	< 0.0001	Significant
2,4-D	395.64	1	395.64	28.26	< 0.0001	
KN	59.65	1	59.65	4.26	0.0457	
PSK	80.82	1	80.82	5.77	0.0211	
2,4-D × KN concentration (conc.)	0.2239	1	0.2239	0.0160	0.9000	
2,4-D × PSK conc.	0.2239	1	0.2239	0.0160	0.9000	
KN × PSK conc.	72.54	1	72.54	5.18	0.0284	
2,4-D × conc.²	103.66	1	103.66	7.40	0.0097	
KN × conc.²	386.71	1	386.71	27.62	< 0.0001	
PSK × conc.²	196.42	1	196.42	14.03	0.0006	
2,4-D × KN × PSK conc.	306.49	1	306.49	21.89	< 0.0001	
2,4-D² × PSK conc.	51.76	1	51.76	3.70	0.0618	
Residual	546.04	39	14.00			
Lack of fit	28.35	3	9.45	0.6572	0.5837	Non- significant
Pure error	517.68	36	14.38			
Cor total	3151.12	50				
SD	3.74	R²	0.8267			
Mean	12.85	Adjusted R²	0.7778			
CV%	29.12	Predicted R²	0.7089			
PRESS	917.16	Adeq Precision	14.0482			
Model type^b^	Reduced cubic model					

Model statistics and estimated coefficients for regression are included

^a^Response = 28.00 − 27.94 2,4-D + 20.13 KN − 445.0 PSK + 12.22 2,4-D^2^ conc. – 27.75 KN^2^ conc. + 1977 PSK^2^ conc. + 9.272 2,4-D × KN conc. + 273.0 2,4-D × PSK conc. + 212.5 KN × PSK conc. – 190.6 2,4-D × KN × PSK conc. – 116.8 2,4-D^2^ × PSK conc

^b^Forced terms: 2,4-D; KN; PSK; Removed: 2,4-D^2^ × KN conc., 2,4-D × KN^2^ conc., KN^2^ × PSK conc., 2,4-D × PSK conc.^2^, KN × PSK^2^ conc

The results of varied medium supplements viz. 2,4-D, KN, and PSK on micro-callus formation from the dividing protoplasts of *A. gigas* is presented in Table 3 and results revealed that there is linear interaction of the tree factors according to the RSM model. There was a significant interaction between the varied factors 2,4-D × KN × PSK, 2,4-D^2^ × KN, 2,4-D^2^ × PSK, and 2,4-D and KN^2^. The results of the 2D contour plot and 3D response surface plots are presented in Fig. 3C and D. The optimized results for microcolony formation from the dividing protoplasts were 0.5 mg L^− 1^ 2,4-D + 0.9 mg L^− 1^ KN and 90 nM PSK. The above results depicted the role of PSK and its interaction with 2,4-D and KN in microcolony formation from dividing protoplasts.

**Table 3:** ANOVA table for the effect 2,4-D, KN, and PSK on the microcolony formation of *Angelica gigas* protoplasts after 4 weeks of culture

Source	Sum of Squares	df	Mean Square	F value	*p* value Prob > F	Significance
Model^a^	4258.24	12	354.85	10.00	< 0.0001	Significant
2,4-D	89.55	1	89.55	2.52	0.1205	
KN	1160.59	1	1160.59	32.70	< 0.0001	
PSK	139.92	1	139.92	3.94	0.0543	
2,4-D × KN concentration (conc.)	0.2239	1	0.2239	0.0063	0.9371	
2,4-D × PSK conc.	108.36	1	108.36	3.05	0.0887	
KN × PSK conc.	340.52	1	340.52	9.59	0.0037	
2,4-D × conc.²	138.18	1	138.18	3.89	0.0558	
KN × conc.²	22.73	1	22.73	0.6405	0.4285	
2,4-D × KN × PSK conc.	376.34	1	376.34	10.60	0.0024	
2,4-D² × KN conc.	571.74	1	571.74	16.11	0.0003	
2,4-D² × PSK conc.	395.64	1	395.64	11.15	0.0019	
2,4-D × KN² conc.	541.79	1	541.79	15.26	0.0004	
Residual	1348.79	38	35.49			
Lack of fit	73.28	2	36.64	1.03	0.3659	Non-significant
Pure error	1275.51	36	35.43			
Cor total	5607.04	50				
SD	5.96	R²	0.7594			
Mean	29.68	Adjusted R²	0.6835			
CV%	20.07	Predicted R²	0.6310			
PRESS	2069.21	Adeq Precision	9.8897			
Model type^b^	Reduced cubic model					

Model statistics and estimated coefficients for regression are included

^a^Response = 45.39–60.17 2,4-D – 4583 KN – 282.0 PSK + 28.61 2,4-D conc.^2^ – 36.17 KN conc.^2^ + 125.7 2,4-D × KN conc. + 646.46 2,4-D × PSK conc. + 309.1 KN × PSK conc. – 211.2 2,4-D × KN × PSK conc. – 38.80 2,4-D^2^ × KN conc. – 322.8 2,4-D^2^ × PSK conc. – 56.66 2,4-D × KN^2^ conc

^b^Forced terms: 2,4-D; KN, PSK; Removed: PSK^2^ conc., KN^2^ × PSK conc., 2,4-D × PSK^2^ conc., KN × PSK^2^ conc

The ANOVA results of the effect of 2,4-D, KN, and PSK on induction of embryogenic callus from micro-calli of *A. gigas* are presented in Table 4. The results showed that significant interaction between two factors 2,4-D and KN. KN × PSK, the secondary effect of PSK^2^, the interaction of independent factors viz. 2,4-D × KN × PSK, 2,4-D^2^ × KN, and 2,4-D^2^ × PSK were significant according to the RSM model. The 2D contour plot and 3D response surface plots are presented in Fig. 3E and F. The overall results depicted that the residual effect of PSK is there on the formation of embryogenic callus from micro-calli *A. gigas*. The optimal conditions for embryogenic callus induction were 1.5 mg L^− 1^ 2,4-D and 1 mg L^− 1^ KN.

**Table 4:** ANOVA table for the effect of 2,4-D, KN, and PSK on embryogenic callus formation of *Angelica gigas* protoplasts after 12 weeks of culture

Source	Sum of Squares	df	Mean Square	F value	*p* value Prob > F	Significance
Model^a^	8173.77	11	743.07	19.98	< 0.0001	Significant
2,4-D	381.63	1	381.63	10.26	0.0027	
KN	10.67	1	10.67	0.2868	0.5953	
PSK	560.67	1	560.67	15.08	0.0004	
2,4-D × KN concentration (conc.)	2223.37	1	2223.37	59.79	< 0.0001	
2,4-D × PSK conc.	9.38	1	9.38	0.2521	0.6184	
KN × PSK conc.	165.38	1	165.38	4.45	0.0414	
2,4-D × conc.²	68.16	1	68.16	1.83	0.1836	
PSK × conc.²	724.16	1	724.16	19.47	< 0.0001	
2,4-D × KN × PSK conc.	737.04	1	737.04	19.82	< 0.0001	
2,4-D² × KN conc.	350.21	1	350.21	9.42	0.0039	
2,4-D² × PSK conc.	1725.21	1	1725.21	46.39	< 0.0001	
Residual	1450.27	39	37.19			
Lack of fit	12.72	3	4.24	0.1062	0.9559	Non-significant
Pure error	1437.56	36	39.93			
Cor total	9624.04	50				
SD	6.10	R²	0.8493			
Mean	11.14	Adjusted R²	0.8068			
CV%	54.75	Predicted R²	0.7130			
PRESS	2762.36	Adeq Precision	17.3146			
Model type^b^	Reduced cubic model					

Model statistics and estimated coefficients for regression are included

^a^Response = 25.37–36.78 2,4-D – 10.67 KN – 613.8 PSK + 13.65 2,4-D^2^ conc. + 3571 PSK^2^ conc. – 5.111 2,4-D × KN conc. + 1176 2,4-D × PSK conc. + 116.7 KN × PSK conc. – 295.6 2,4-D × KN × PSK conc. + 30.37 2,4-D^2^ × KN conc. – 674.1 2,4-D^2^ × KN conc

^b^Forced terms: 2,4-D; KN, PSK; Removed: KN^2^ conc., 2,4-D × KN^2^ conc. KN^2^ × PSK conc., 2,4-D × PSK^2^ conc., KN × PSK^2^ conc

### Plant regeneration from protoplast-derived embryogenic callus

**Fig. 4:**
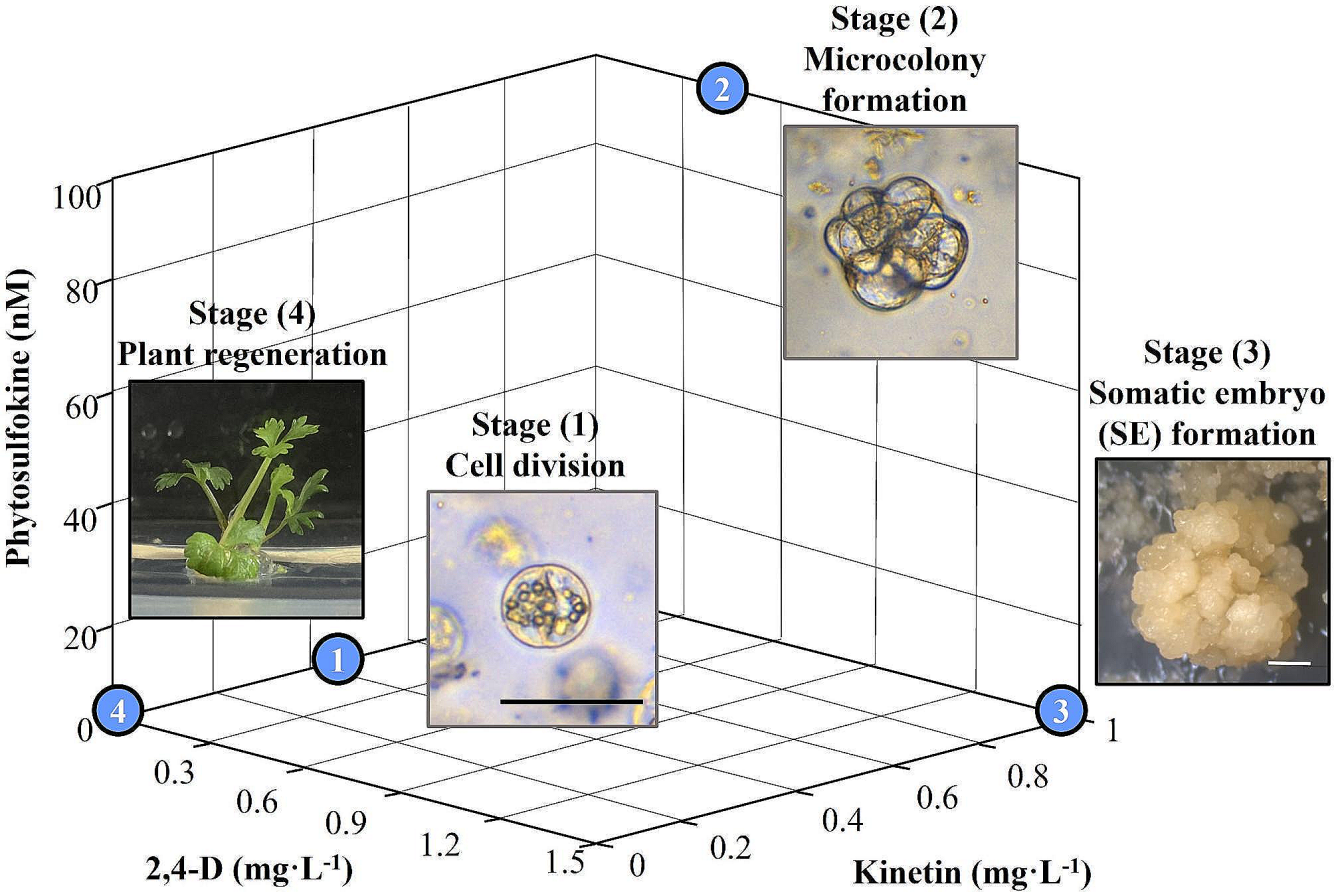
Graphical representation optimized concentration of three factors (2,4-D, KN, and PSK) for protoplast division (stage 1), microcolony formation (stage 1), induction embryogenic callus (stage 3), and plant regeneration (stage 4). Scale bars: 50 μm (black bars) and 2 mm (white bars)

**Fig. 5:**
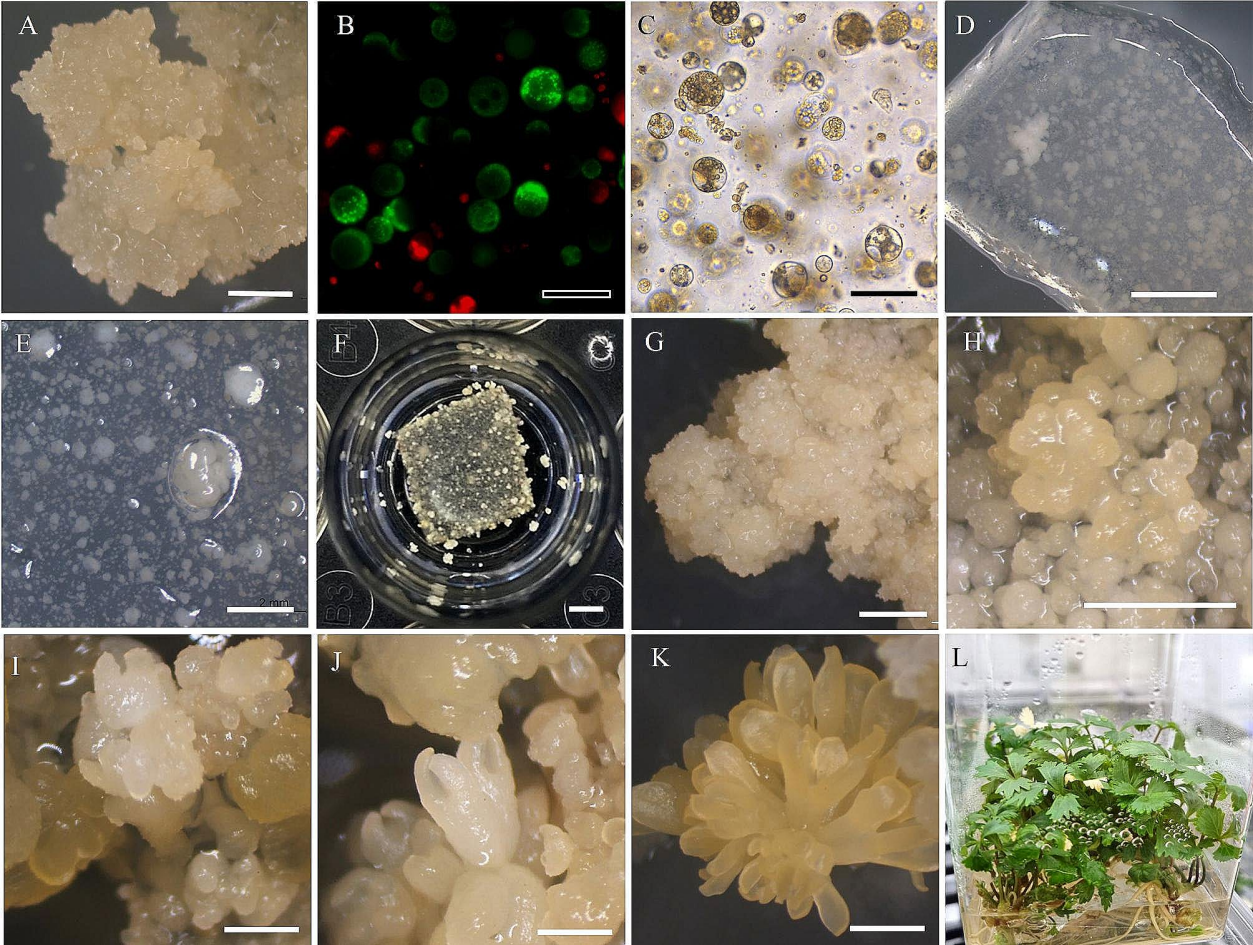
Protoplast isolation, culture using the TAL method, division of protoplasts, micro-callus formation, development of embryos, and plant regeneration in *Angelica gigas*. The cultures were maintained in the dark. Embryogenic callus used for protoplast isolation (**A**), fluorescence microscopic images – green fluorescence represents viable protoplasts stained using FDA and represents dead protoplasts stained with PI (**B**), protoplasts embedded in TAL after 3 days of culture (**C**), micro-calli in TAL cultures after 9 weeks of culture (**D**), micro-calli formed in TAL cultures after 10 weeks of culture (**E**, **F**). Embryogenic callus (**G**), globular (**H**), heart (**I**), and cotyledonary staged embryos (**J, K**) developed from protoplast regenerated callus after 8–10 weeks of sub-culture of callus on MS semi-solid medium, plants regenerated by germination of embryos on MS semi-solid medium (**L**). Scale bars: 100 μm (black bars) and 2 mm (white bars)

**Fig. 6:**
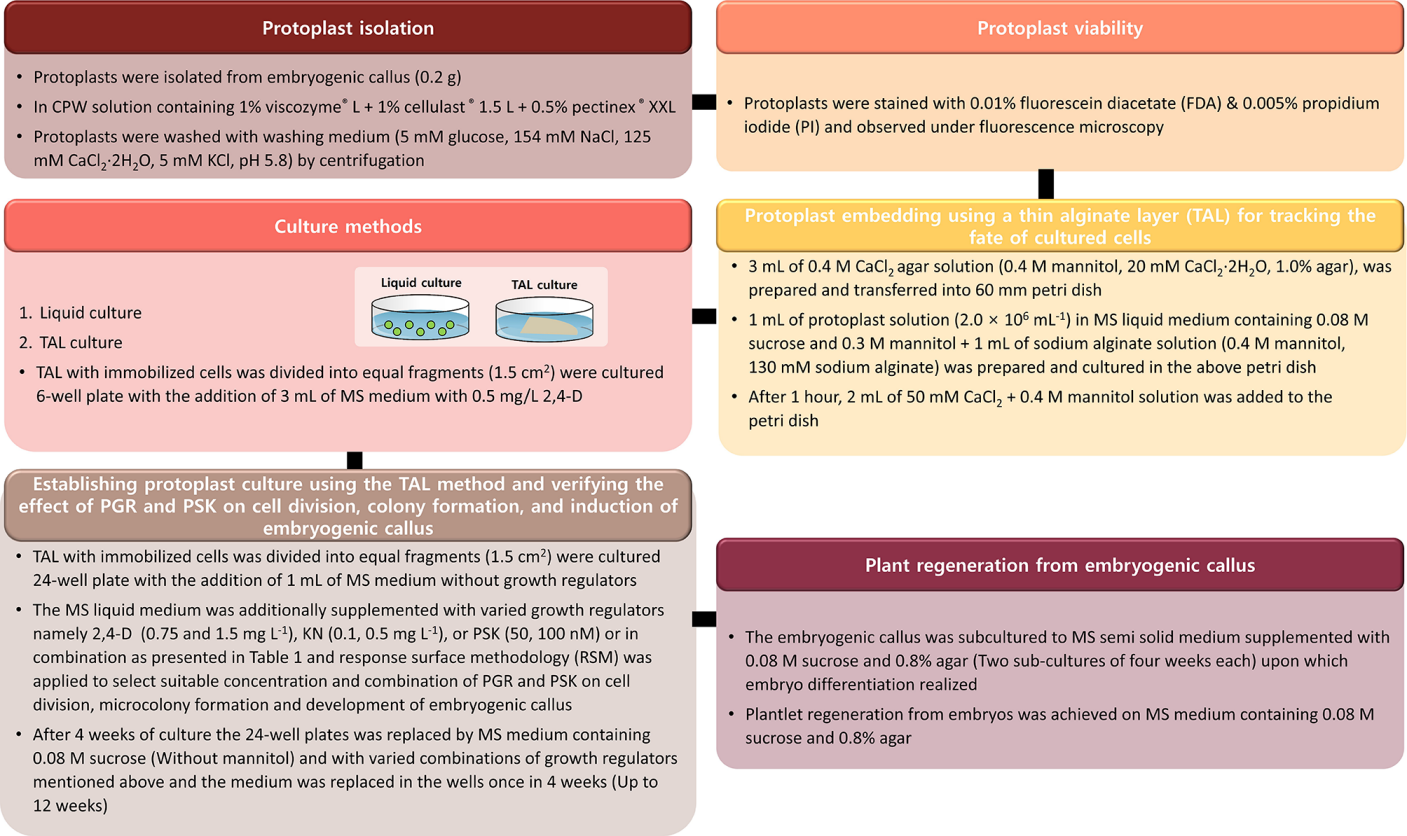
Flow chart illustrating a step-by-step approach for protoplast isolation, culture, and plant regeneration of *Angelica gigas*

The embryogenic callus was separated from TAL and sub-cultured twice at an interval of four weeks on MS semi-solid medium containing 0.08 M sucrose and 0.8% agar resulting in embryo development. Matured embryos were sub-cultured to MS semi-solid medium containing 0.08 M sucrose and 0.8% agar resulting in germination and plantlet development. The optimized medium conditions of protoplast division, microcolony formation, induction of embryogenic callus, and plant regeneration are presented in the coordinate plane in Fig. 4. Figure 5 shows pictures of the entire series of events, beginning with the isolation of protoplasts and TAL cultures, differentiation of embryos from embryogenic callus, embryo maturation, and plant regeneration. Protoplasts of *A. gigas* were isolated from embryogenic callus (Fig. 5A) and cultured in liquid and TAL medium, the viability of protoplasts was observed under fluorescent microscopy after fluorescein diacetate (FDA) and propidium iodide (PI) staining, protoplasts emitting green fluorescence were viable and protoplasts emitting red fluorescence were dead cells (Fig. 5B). TAL embedded protoplasts (Fig. 5C) were involved in division and formed micro-calli, protoplast derived micro-calli with TAL cultures after 9 weeks of culture and after 10 weeks are presented in Fig. 5D and E-F respectively. Subsequent culture of embryogenic callus (Fig. 5G) has led to the induction of globular embryos (Fig. 5H), heart-staged embryos (Fig. 5I), matured embryos (Fig. 5J and K), and regenerated into plants after embryo transplantation to semi-solid medium (Fig. 5L). The schematic diagram of overall comprehensive protoplast cultures and plant regeneration is presented in Fig. 6.

## Discussion

Protoplasts derived from somatic cells upon culture can be involved in division, differentiation, callus formation, and regeneration into whole plants since plant cells have an amazing capacity to exhibit cellular totipotency [31, 32]. Protoplasts are ideal tools for varied studies in the fields of physiology, genetics molecular biology [1, 2]. Protoplasts are utilized in understanding the key processes involved in cell wall recovery, cell cycle re-entry, callus formation, and expression of totipotency [32, 33]. Protoplast fusion techniques are quite useful in overcoming breeding barriers and producing cytoplasmic and somatic hybrids with desirable characteristics [1, 2, 4]. Protoplast technology has been recently utilized for genetic engineering and genome editing in plants. Genome-edited single-cell clones have been produced as a result of technologies such as transiently expressing genes in plant protoplasts and genome-editing protoplasts utilizing the CRISPR/Cas9 system, which has resulted in the regeneration of entire plants [5, 34]. To achieve all these processes protoplast-to-plant regeneration should be thoroughly established. Even though protoplast regeneration methods have been developed in several plant species, plant regeneration is still a bottleneck in several plant species [1, 5].

*Angelica gigas* is an important medicinal plant that has many therapeutic values [16], earlier we have reported successful protoplast isolation using the embryogenic callus of *A. gigas* and reported the optimum duration of enzyme treatment, medium for culture of protoplasts and plant regeneration [19]. In the present study, we focused on the protoplast culture methods and plant growth regulators and the application of PSK for the promotion of cell division, callus induction, and plant regeneration. In addition, we applied the RSM methodology to specify the concentration of 2,4-D, KN, and PSK on induction of protoplast division, microcolony formation, and induction of somatic embryogenic callus. We tracked the fate of protoplasts in culture for their initial growth, development, and division by embedding the protoplasts in a TAL, the results showed only 6.35% of cells were involved in the division after 3 days in culture, even though the majority of protoplasts involved in expansion and elongation, they did not involve in the division. Similar to the current results, Chupeau et al. [35] made a closer observation of early morphological changes via time-lapse imaging of *Arabidopsis* protoplasts and recorded first cell division on day 4 after culture. In addition, Sakamoto et al. [36] described the elongation of cultured protoplasts of *Arabidopsis* in 1–3 days, and many protoplasts were divided on days 6 or 7. Furthermore, Sheahan et al. [37] recorded that the protoplasts that possessed larger vacuoles were involved in the division. Marthy [38], and Cosgrove [39] believe that larger vacuoles in the cells are responsible for turgor pressures and are the driving force for elongation, growth, and subsequent division of the cells.

Protoplasts are usually cultured in a liquid medium since this culturing method is a simpler and easier technique. Protoplasts cultured in a liquid medium are involved in regaining the cell wall, cell division, and callus formation, however, in the majority of cases, regeneration of plants from the callus has failed due to cell aggregation-induced cell death [1, 40, 41]. To overcome the limitation of liquid culture, several studies have developed protoplast immobilization and protoplast embedding methods have followed using agarose or alginate [1, 42]. In the present study, we cultured the protoplasts of *A. gigas* by using both a liquid medium and the TAL method and compared the methods for protoplast division, 2–3 celled stage, and microcolony formation on 3, 5, and 7 weeks of culture. The results revealed that the liquid medium was not ideal, even though protoplasts remained viable for several weeks, they were not involved in division. However, with TAL cultures, protoplasts were involved in the division, 2.66% of protoplasts reached the 2-3-celled stage, and 12.20% were in the microcolony stage. The TAL method was developed by Golds et al. [43] and alginate-embedding methods have been widely used for protoplast culture, callus induction, and plant regeneration in many plant species including *Arabidopsis thaliana*, *Beta vulgaris*, *Daucus carota*, *Nigella damascena*, *Platycodon grandifloras*, and *Nicotiana tobacco* [30, 44–47]. Alginate-based hydrogels have been reported to immobilize the protoplast efficiently and facilitate the immobilized protoplasts to regain the cell walls, cell division, and callus formation [1, 5].

Standard protocols for cultivating protoplasts in a liquid medium result in limited proliferation activity and cell death, which makes them less successful in regenerating tissue. The approach known as TAL was created to solve the problems with protoplasts [1]. By crosslinking with divalent alkaline metal ions, such as calcium, the alginate creates a hydrogel. Alginate hydrogels can immobilize protoplasts while preserving their viability without causing cell aggregation [48]. Additionally, immobilization of protoplasts in TAL reduces the synthesis of polyphenols, which may improve cell division, sustain cell wall renewal, and extend cell viability [1]. Furthermore, TAL embedding will supply additional calcium and aid in the cultured protoplasts’ ability to resynthesize their cell walls and participate in the protoplast division [44]. An additional benefit of TAL cultures over liquid cultures is that they assist the extra calcium ions, as opposed to agarose embedding, which is in charge of facilitating the extra potassium ions. According to a report, protoplasts embedded before culture—particularly those immersed in an alginate matrix—generally exhibited a higher rate of colony formation than protoplasts that were not embedded. The control of lipid peroxidation, which stabilizes the membrane, the prevention of cell precursor and other metabolite leakage, and the reduction of ethylene levels could all contribute to the beneficial effects of embedding on cells [49]. Furthermore, TAL permits the fast supply of gasses and growth factors to the embedded cells, including metabolites, hormones, and signaling molecules [1]. In addition to increasing plating efficiency, protoplast immobilization in TAL is necessary for mitotic activity by protoplast-derived cells [50]. A general conclusion was drawn from data from numerous publications, indicating that colony formation is more common in thinner matrices, most likely due to increased compound diffusion from the medium to the protoplasts [2, 44, 50, 51].

The crucial step in protoplast cultures is enhancing the protoplast division with the cultures, cell proliferation, callus induction, and subsequent plant regeneration [1, 5]. Recently, varied growth supplements have been tested along with plant growth regulators to achieve good cell division, and cell proliferation during protoplast cultures. Polyamines and peptide growth factors such as PSK have been successfully used as growth additives. Therefore, in the current studies, we utilized PSK (0, 50, 100 nM), along with 2,4-D (0, 0.75, 1.5 mg L^− 1^) and KN (0, 0.5 and 1.0 mg L^− 1^) and applied RSM methodology to find out optimum concentrations which can promote cell division, cell proliferation, micro-callus, and embryogenic callus induction. The results showed that according to CCD responses, the ideal concentration of protoplast division was 0.04 mg L^− 1^ 2,4-D + 0.5 mg L^− 1^ KN + 2 nM PSK as determined after 4 weeks in TAL cultures. The optimized concentrations were 0.5 mg L^− 1^ 2,4-D + 0.9 mg L^− 1^ KN and 90 nM PSK during micro-callus formation. These results display the role of PSK and its interaction with 2,4-D and KN in initiating cell division and cell proliferation to form micro-callus in *Angelica gigas* protoplast cultures. Similar, to the current results PSK has been successfully used along with other plant growth regulators to achieve excellent protoplast division and cell proliferation and differentiation in *Brassica oleracea* var. *capitata* [22], *Daucs* [23], *Fagopyrum tataricum* [25], *Nigella damascena* [45]. We also tested the impact of PSK, 2,4-D, and KN concentrations on embryogenic callus regeneration from protoplast-derived micro-callus after 12 weeks of cultures by RSM, and results depicted that 1.5 mg L^− 1^ 2,4-D and 1 mg L^− 1^ KN was excellent for induction embryogenic callus. These results demonstrate that the 2,4-D and KN combination alone is enough for the induction of embryogenic callus from the protoplast-derived micro-callus in *Angelica gigas*. In contrast to these results, PSK was found to stimulate the induction of somatic embryogenesis in *Daucus carota* [51], and *Crytopmeria japonica* [52]. In *Angelica gigas* protoplast culture we successfully applied and utilized CCD and RSM in predicting the concentrations of PSK and 2,4-D and KN concentrations which are responsible for the protoplast division, cell proliferation, and induction of somatic embryogenic callus. In concurrence with these results, RSM has been applied efficiently during protoplast cultures of other species and interpreting the key factors responsible for the protoplast division, cell proliferation, callus formation, and pant regeneration [30, 53].

The promising intercellular signaling molecule PSK, a peptidyl plant growth factor, has been found to participate in division in protoplast cultures, increase cellular proliferation, and differentiate cells [23]. Enhanced protoplast division, which results in the production of calluses and embryogenic masses, has been demonstrated in several species when PSK is added to the culture media. Li et al. [26] and Shen et al. [54] have clarified the potential mechanism of interaction with PSK with its receptors of the plasma membrane and subsequent signal transduction, gene expression, and cell growth and development. PSK will attach to its receptors at the cell surface known as PSK receptor proteins (PSKRs) during PSK signal transduction. Research has demonstrated that PSKR1 in *Arabidopsis thaliana* possesses a calmodulin (CaM) binding site in addition to a cyclic guanylate kinase domain [55, 56]. As a result, PSK and PSKR interaction encourages cGMP production, which in turn triggers downstream signal transduction. The interplay of potassium ions during the transmission of PSK signals during *Arabidopsis thaliana* protoplast cultures was elucidated by Stuhrwohlt et al. [57]. Furthermore, Talke et al. [58] showed how cGMP regulates cyclic nucleotide-gated channels (CNGC), and in particular, how CNGC17 stimulates the growth of protoplasts in plant cells. Further evidence of the roles played by BRI (brassinolide insensitive1) factors, AHA1 (activator of Hsp90 ATpase1), AHA2, and H^+^-ATPases during signal transduction was provided by Ladwing et al. [59]. It was recently reported by Hu et al. [60] that the PSKR1 protein activity is governed via the ubiquitin/protease degradation pathway. PSK singling, in conjunction with other phytohormones, facilitates the regulation of protoplast development, division, and subsequent morphogenic processes. However, the precise mechanism of PSK signal transduction is still unknown.

The embryogenic callus of *Angelica gigas* was subcultured to MS basal semi-solid medium without growth regulators upon which embryos involved differentiation and developed into cotyledonary embryos in 8–10 weeks of subculture. Cotyledonary embryos germinated and developed into plantlets on an MS basal semi-solid medium. This successful step-wise plant regeneration via protoplast culture is useful for the improvement of *Angelica gigas* with the application of biotechnological methods.

The main innovations of the current findings are the successful division of protoplasts, micro-callus development, induction of embryogenic callus, somatic embryogenesis, and plant regeneration in *A. gigas* by the optimization of critical variables through the use of RSM. Our next goals include identifying genes of interest that are unique to dividing cells and embryogenic cells, as well as transcriptome changes in the protoplast-derived *A. gigas* embryogenic callus. Ultimately, we hope to produce genome-edited plants.

## Conclusion

The application of CCD-RSM for optimization of growth factors and plant growth regulators during protoplast division, cell proliferation, micro-callus, and induction of embryogenic callus stages resulted in the development of an effective and repeatable protoplast-to-plant regeneration method in *Angelica gigas*. The overall technique consists of several phases, such as employing cell wall-degrading enzymes to isolate protoplasts from embryogenic callus, purifying protoplasts, and cultivating them using the TAL method. Supplying specific quantities of PSK, 2,4-D, and KN to achieve ideal protoplast division, cell proliferation, micro-callus development, and embryogenic callus regeneration. Plant regeneration and embryo induction subsequently make this process repeatable (Schematic diagram – Fig. 6). This protocol can be used for fundamental studies, genetic transformation, and improvement of this medicinal plant using genome editing technologies.

## Methods

### Plant material

Embryogenic callus of *Angelica gigas* Nakai which was induced on MS [61] medium supplemented with 3% (w/v) sucrose and 1.0 mg L^− 1^ 2,4-D was used in the present studies [19].

### Protoplast isolation

Protoplasts were isolated from 0.2 g of embryogenic callus of *Angelica gigas* using 2 mL of cell protoplast washing (CPW) medium [CaCl_2_.2H_2_O, 0.4 M mannitol, 20 mM of 2-(N-morpholino) ethanesulfonic acid (MES), pH 5.8, filter sterilized] containing 1.0% Viscozyme^®^ L + 1% Celluclast^®^ 1.5 L + 0.5% Pectinex^®^ XXL (Novaozymes, Bagsvaerd, Denmark) taken in 60 mm petri plates. The Petri plates containing enzymatic solution were incubated for 7 h on a shaking incubator at 40 rpm under dark conditions and at 24 ± 1^o^C. Subsequently, the protoplast suspension was filtered using a 40 μm nylon mesh sieve (Merck, Darmstadt, Germany) and the solution was centrifuged at 100 × *g* for 5 min at room 24 ± 1 ^o^C. The pellet was resuspended in W5 protoplast washing medium (5 mM glucose, 154 mM NaCl, 125 mM CaCl_2_.2H_2_O, 5 mM KCl, pH 5.8) and centrifuged again for 5 min as above. Subsequently, protoplasts were suspended in 0.4 M mannitol solution and protoplast density was counted using a hemocytometer (Sigma, St. Louis, USA) and optical microscope (DMi8, Leica, Wetzler, Germany).

### Analysis of the viability of protoplasts

Protoplasts were stained using 0.01% FDA (596-09-8; Sigma-Aldrich) and 0.005%, PI (25535-16-4; Sigma-Aldrich), then observed under fluorescence microscopy (DMi8, Leica, Wetzler, Germany) and using Leica Application Suite X 6.4v software. The number of living, and dead protoplast, and initial division of protoplasts until micro-callus formation stages were tracked and quantified. Based on FDA/PI staining the percentage of viable protoplasts was calculated as follows: viable protoplasts = (number of stained protoplasts/ total number of protoplasts) x 100 [62].

### Analysis of protoplast for growth, and development

Initially, protoplasts were cultured using the TAL method [1]. In the first step, 3 mL of 0.4 CaCl_2_ agar solution was prepared (0.4 M mannitol, 20 mM CaCl_2_.2H_2_O, 1.0% agar) and transferred into 60 mm Petri plates. After 30 min, 1 mL of protoplast solution (2.0 × 10^6^ mL^− 1^) in MS liquid medium containing 0.08 M sucrose and 0.3 M mannitol, and 1 mL of sodium alginate solution (0.4 M mannitol, 130 mM sodium alginate) was prepared these two solutions were mixed properly and added the Petri plates which were prepared earlier. After another one-hour lapse of time, 2 mL of 50 mM CaCl_2_ with 0.4 M mannitol solution was added to the top of the Petri plates.

### Protoplast culture

Protoplasts were adjusted to 1 × 10^6^ cells per mL^− 1^ and were cultured by using two different methods. Firstly, protoplasts were cultured using the conventional liquid culture method in MS liquid medium supplemented with 0.3 M mannitol, 0.08 M sucrose, and 0.5 mg L^− 1^ 2,4-D in 10 mm Petri dishes. In the second method, protoplasts were immobilized in a TAL [1], and then TAL were cut into (1.5 cm^2^) sections and were cultured in a 6-well plate (SPL Ltd., Pocehon, Republic of Korea) containing 3 mL of MS liquid medium supplemented with 0.3 M mannitol, 0.08 M sucrose, and 0.5 mg L^− 1^ 2,4-D. The observation was made on percentage cell division, 2–3 cell stage, and microcolony stage after 3, 5, and 7 weeks after culture. Among all the culture methods, TAL cultures yielded better results viz. cell division, and microcolony formation, therefore, in the subsequent experiment to optimize growth regulators/growth factors concentration TAL method was followed.

### Application of RSM to optimize the 2,4-D, KN, PSK on cell division, microcolony formation, and embryogenic callus induction

The protoplasts were cultured using the TAL method as above, and then TAL fragments (1.5 cm^2^) were cultured in 24 well plates by using 1 mL of MS liquid medium supplemented with 0.3 M mannitol, and 0.08 M sucrose, without a growth regulator used as control (designated as 0). The MS liquid medium was additionally supplemented with various growth regulators namely 2,4-D (0, 0.75, 1.5 mg L^− 1^), 2,4-D in combination with KN (0, 0.5, and 1.0 mg L^− 1^), and PSK (0, 50, 100 nM) on protoplast division and microcolony/micro-calli formation (Table 1).

We employed CCD-RSM to determine optimal conditions for protoplast division, microcolony formation, and development of embryogenic callus, after one week, four weeks, and twelve weeks of protoplast culture respectively. Design-Expert software (Version 13; Stat-Ese Inc., Minneapolis, MN, USA) was used for CCD modeling, and to assess the significance of data and interactions by ANOVA followed by the Fisher test. Each experimental design consisted of 3 replicates across 17 designed runs in the CCD design, with the center point set to 3. The space type was designed with 3 centers, 6 axial points, and 8 factorials. The results were presented in ANOVA tables for each set of experiments viz. cell division (Table 2), microcolony formation (Table 3), embryogenic callus induction (Table 4), a two-dimensional contour plot, and three-dimensional 3D models.

### Plant regeneration

The embryogenic calli-containing embryos were sub-cultured to MS semi-solid medium with 0.08 M sucrose and 0.8 agar and maintained for 8 weeks by sub-culturing callus once every four weeks, upon which embryos were developed on the callus, and involved in maturation. Matured embryos germinated and developed into plantlets upon sub-culturing to fresh MS medium containing 0.08 M sucrose and 0.8 agar in another two weeks. At this stage, the cultures were maintained under 14 h light and 8 h dark photoperiod with a light intensity of 40 µmol m^− 2^s^− 1^.

## Data Availability

The raw data and materials are available with Dr. So-Young Park, Department of Horticulture, Chungbuk National University, Cheongju 28644, Republic of Korea.
